# Large‐scale production, purification, and function of a tumor multi‐epitope vaccine: Peptibody with bFGF/VEGFA

**DOI:** 10.1002/elsc.202000020

**Published:** 2020-07-29

**Authors:** Ligang Zhang, Chengcheng Jiang, Xi Chen, Jiangtao Gu, Qifang Song, Hui Zhong, Sheng Xiong, Qingfeng Dong, Jin‐Chen Yu, Ning Deng

**Affiliations:** ^1^ Guangdong Province Engineering Research Center for Antibody Drug and Immunoassay Department of Biology Jinan University Guangzhou P. R. China; ^2^ The Biomedicine Translational Institute in Jinan University Guangzhou P. R. China; ^3^ Guangdong Jida Genetic Medicine Engineering Research Center Co. Ltd Guangzhou P. R. China; ^4^ Bio‐Thera Solution Co. Ltd Guangzhou P. R. China

**Keywords:** bFGF/VEGFA, fermentation, Peptibody, purification, tumor angiogenesis

## Abstract

In tumor tissue, basic fibroblast growth factor (bFGF) and vascular endothelial growth factor A (VEGFA) promote tumorigenesis by activating angiogenesis, but targeting single factor may produce drug resistance and compensatory angiogenesis. The Peptibody with bFGF/VEGFA was designed to simultaneously blockade these two factors. We were aiming to produce this Fc fusion protein in a large scale. The biological characterizations of Peptibody strains were identified as *Escherichia coli* and the fermentation mode was optimized in the shake flasks and 10‐L bioreactor. The fermentation was scaled up to 100 L, with wet cell weight (WCW) 126 g/L, production 1.41 g/L, and productivity 0.35 g/(L·h) of IPTG induction. The target protein was isolated by cation‐exchange, hydrophobic and Protein A chromatography, with total recovery of 60.28% and HPLC purity of 86.71%. The host cells protein, DNA, and endotoxin residues were within the threshold. In mouse model, immunization of Peptibody vaccine could significantly suppressed the tumor growth and angiogenesis, with inhibition rate of 57.73 and 39.34%. The Peptibody vaccine could elicit high‐titer anti‐bFGF and anti‐VEGFA antibodies, which inhibited the proliferation and migration of Lewis lung cancer cell cells by decreasing the Akt/MAPK signal pathways. Therefore, the Peptibody with bFGF/VEGFA might be used as a therapeutic tumor vaccine. The large‐scale process we developed could support its industrial production and pre‐clinical study in the future.

**Abbreviations**
bFGF: basic fibroblast growth factor
DO: dissolved oxygen
*E. coli*: *Escherichia coli*
HCP: host cells protein
IHC: immunohistochemistry
LL‐2: Lewis lung cancer cell
qRT‐PCR: real‐time quantitative PCR
SEM: scanning electron microscope
VEGFA: vascular endothelial growth factor A
WCW: wet cell weight

## 1 INTRODUCTION

In the development of solid tumor, basic fibroblast growth factor (bFGF) and vascular endothelial growth factor A (VEGFA) can promote tumor progression and angiogenesis in autocrine and paracrine manners [1]. These two factors combined with their cognate receptors play key roles in the proliferation, metastasis and differentiation of tumor cells and vascular endothelial cells [2]. Moreover, there exists a synergistic effect between bFGF and VEGFA to form new vessels through platelet‐derived growth factor signal pathway [3]. It has been demonstrated that tumorigenesis will be withdrawn if the signal pathways of tumor angiogenesis are blockaded [4]. Thus, angiogenesis inhibition may be a viable treatment approach for the highly vascular tumor types, such as ovarian cancer, lung cancer and breast cancer [5, 6, 7]. However, targeting single factor may produce drug resistance and compensatory angiogenesis because the roles of other factors may be strengthened [8]. Several inhibitors of growth factor, receptor and kinases such as Bevacizumab, Ramucirumab, Aflibercept, Sunitinib, Sorafenib, and Pazopanib have been brought to clinical treatment for malignant tumors but most of these approaches are based on the physiological effects of single pathway, which may be insufficient to induce enduring anti‐tumor efficacy [9]. There is a great need to improve current therapy by exploring combinatorial strategies [10]. Therefore, blockade of bFGF and VEGFA in a simultaneous manner may be an effective strategy to inhibit tumorigenesis and tumor angiogenesis.

The classical recombinant peptide vaccine are comprised of several epitopes and recognized by only helper T cell or cytotoxic T cell. Nowadays, the novel peptide drugs contain antigenic epitopes of B cell and T cell in order to elicit specific humoral and cellular responses [11]. We selected three antigenic epitopes from human bFGF and three antigenic epitopes from human VEGFA through phage display and bioinformatic prediction [12]. Moreover, Fc domain of IgG can fuse to recombinant peptides, which is well‐known as a promising platform with FDA approval [13]. The low molecular weight and fast renal clearance will cut down the serum half‐life of peptide drugs [14]. The addition of Fc domain will protect the peptide from lysosomal breakdown to increase the half‐life and therapeutic activity, making it a promising strategy for tumor therapy [15]. Abatacept is a CTLA‐4‐Fc fusion protein to down‐regulate T cells activation in RA and has a half‐life more than 10 days [16]. Fc fusion protein can promote other protein properties, including immunogenicity, efficacy, solubility, and purification [17]. Here, we had applied Fc fusion concept to bFGF/VEGFA epitopes and designed a multi‐epitope vaccine called Peptibody.

**PRACTICAL APPLICATION**

The fed‐batch fermentation process of Peptibody strains we developed was scaled up from flask to 100‐L bioreactor, supporting the industrial production for its pre‐clinical study in the future. We shed light on the functional aspects of Fc fragment in Protein A affinity chromatography, which would allow devising better procedures for Peptibody purification. Peptibody fusion protein might be used as a potential therapeutic tumor vaccine for the inhibitory effects on the tumor growth, migration and angiogenesis in vitro and vivo experiments.

*Escherichia coli* (*E. coli*) with recombinant plasmid is a versatile experimental, medical and industrial bacterium for heterologous protein production, which grows rapidly to a high cell density with inexpensive carbon sources [18]. The inducible T7 promoter with lac operon is a paradigm for transcriptional regulation in *E. coli*, which can be induced to its full strength by IPTG, a structural non‐metabolizable analogue of allolactose [19]. Since the bacterial growth and protein expression are fluctuant, optimal culture conditions should be established for fermentation in a large scale, making yields considerable. In the purification procedures, one of the reasons we add Fc domain to the fusion protein is for downstream purification, which can be captured by Protein A chromatography with high affinity [20]. However, the binding will not occur if there are excessive *E. coli* endogenous proteins in the supernatant of cell lysate. Moreover, metal ions are banned from human vaccine; thus, columns chelated metal ions are out of our consideration [21]. In the industrial production, cation‐exchange chromatography, an inexpensive column with high capacity, is widely used to catch recombinant protein with positive charge from soluble fractions and other endogenous proteins with negative charge will flow through in this column [22]. Hydrophobic chromatography is employed for further purification when the hydrophobic groups are exposed in high salinity [23]. At final, the recombinant Peptibody with Fc domain can be successfully captured and purified by Protein A chromatography when most of endogenous protein are removed.

In this study, the immunogenic epitopes from human bFGF/VEGFA and IgG1 Fc domain were inserted into the pET‐28a vector. The best‐performing Peptibody strains were selected from the library and optimized in the shake flasks and 10‐L bioreactor for high level expression. The fed‐batch fermentation of Peptibody was scaled up and conducted in 100‐L bioreactor, followed by bacterial growth and expression regulation. Columns of cation‐exchange, hydrophobic and Protein A chromatography were employed to isolate the target protein and the host cells protein (HCP), DNA and endotoxin residues were further tested. A series of in vivo and in vitro experiments were performed to investigate the inhibitory effects of Peptibody vaccine on tumor progression and angiogenesis. This multi‐epitope Peptibody with bFGF/VEGFA might develop a promising strategy for tumor therapy and the large‐scale process would support its industrial production and pre‐clinical study in the future.

## 2 MATERIALS AND METHODS

### 2.1 Tumor cells and mice

Lewis lung cancer cell (LL‐2) line developed from a primary tumor nodule of the murine Lewis lung cancer model was obtained from the Shanghai Institute of Biochemistry and Cell Biology, Chinese Academy of Sciences. The cells were incubated in DMEM supplemented with 10% FBS, 100 U/mL penicillin and 100 µg/mL streptomycin in a cell incubator of 37℃, 5% CO_2_ and 95% relative humidity. The BALB/c and C57BL/6J mice (female, 6 wk old) were purchased from the Experimental Animals Center of Southern Medical University in Guangzhou, China and housed in the specific pathogen‐free environment. Protocols of animal experiments were conducted and approved by the guidelines of the Institutional Animal Care and Use Committee, Jinan University, Guangzhou, China.

### 2.2 Strain, media, and the biological characterizations test

The oligonucleotide fragments of bFGF/VEGFA‐Fc (Peptibody) were synthesized by Sangon Biotech and inserted into the expression vector pET‐28a. The recombinant plasmid pET‐28a‐Peptibody was transformed into *E. coli* BL21 (DE3) and the Peptibody strains were grown in LB medium containing 10 g/L Tryptone, 10 g/L NaCl, 5 g/L yeast extract and 50 µg/mL Kanamycin for seed activation (37℃, 220 rpm). M9 growth medium containing 15.12 g/L Na_2_HPO_4_·12H_2_O, 3 g/L KH_2_PO_4_, 0.5 g/L NaCl, 1 g/L NH_4_Cl, 0.2 g/L MgCl_2_, 20 g/L Tryptone, 10 g/L yeast extract, 0.01% v/v Glycerol and supplementary medium containing 5 g/L MgCl_2_, 80 g/L Tryptone, 40 g/L yeast extract, 0.2% v/v Glycerol were used for shake‐flask and fed‐batch fermentation. The biological characterizations of Peptibody strains grown in LB medium were identified by long‐time storage, Gram staining, scanning electron microscope (SEM), API 20E and 16S rDNA sequencing.

### 2.3 Optimizations of culture conditions for shake‐flask fermentation

The best‐performing strains were selected out from the library and the fermentation conditions including expression temperature, IPTG concentration, induction time, inoculation density, dissolved oxygen (DO) and IPTG inducing time were optimized in the 500 mL flasks containing 200 mL M9 growth medium (DO 60%). The DO was control according to the ratio between medium volume and the rest flask space. For exploring expression temperature from 20 to 36℃, the other conditions were set IPTG concentration 0.1 mM, induction time 4 h, inoculation density 2%, DO 60% and IPTG inducing time 6 h. For searching IPTG concentration from 0.1 to 0.6 mM, the other conditions were set expression temperature 28℃, induction time 4 h, inoculation density 2%, DO 60% and IPTG inducing time 6 h. For testing induction time from 1 to 6 h, the other conditions were set expression temperature 28℃, IPTG concentration 0.1 mM, inoculation density 2%, DO 60% and IPTG added time 6 h. For testing inoculation density from 1 to 8%, the other conditions were set expression temperature 28℃, IPTG concentration 0.1 mM, induction time 4 h, DO 60% and IPTG inducing time 6 h. For testing DO from 30 to 70%, the other conditions were set expression temperature 28℃, IPTG concentration 0.1 mM, induction time 4 h, inoculation density 2% and IPTG inducing time 6 h. For testing IPTG added time from 2 to 8 h, other conditions were set expression temperature 28℃, IPTG concentration 0.1 mM, induction time 4 h, inoculation density 2% and DO 60%. The expression level of each sample was analyzed by SDS‐PAGE assay and the gel images were scanned to quantify the targeted protein (37.4 kDa) via Bio‐rad Image System (Bio‐rad, USA).

### 2.4 Peptibody production in 10‐L bioreactor

According to the fermentation mode of shake flasks, bioreactor of 10 L (Sartorius, Germany) was employed to the fed‐batch fermentation of Peptibody strains. The bioreactor contained an agitator with three impellers and it was equipped with air flow, temperature, pH, DO, and anti‐foam sensors. There was a panel for operation control and four sets of peristaltic pumps were available for supplies flowing in during fermentation. Initially, Peptibody strains were cultured in a 500 mL flask containing 100 mL LB medium supplemented with 50 µg/mL Kanamycin for 6 h (37℃, 220 rpm). The activated seed strains were inoculated into 5 L M9 growth medium at a density of 2% and the fermentation mode was operated at 37℃, air flow 1.0 vvm and agitation rate 300 rpm. Sparger aeration with filtered air (O_2_ if necessary) and agitation association were employed to maintain DO over 30% saturation. The pH was controlled at 7.0 ± 0.05 via automated addition of hydrochloric acid and ammonia. For avoiding foam development, anti‐foamer was automatically added. The fed‐batch mode was indicated by the sharp loss of DO and commenced by feeding 1.7 L M9 supplementary medium at the speed of 10 mL/min. At the checkpoint of 6 h, IPTG was added into the culture media at the final concentration of 0.1 mM at 28℃. The fermentation was stopped at 11 h and the cells were harvested by contrifugation (8000 rpm, 30 min). Samples of fermentation broth (10 mL) were taken out every 1 h. The values of OD_600_ were detected using a microplate reader (BioTek Instruments, USA). The expression level was analyzed by SDS‐PAGE assay and the gel images were scanned to quantify the targeted protein via Bio‐rad Image System.

### 2.5 Scaling up Peptibody production in 100‐L bioreactor

According to the fermentation mode of 10‐L bioreactor, the fed‐batch fermentation of Peptibody strains was scaled up to 100 L. Initially, Peptibody strains were cultured in five flasks of 500 mL for 6 h (37℃, 220 rpm) and each flask contained 200 mL LB medium supplemented with 50 µg/mL Kanamycin. The activated seed strains (total 1 L) were inoculated into 50 L M9 growth medium at a density of 2% and the fermentation mode was operated at 37℃, air flow 1.0 vvm and agitation rate 300 rpm. Sparger aeration with filtered air (O_2_ if necessary) and agitation association were employed to maintain DO over 30% saturation. The pH was controlled at 7.0 ± 0.05 via automated addition of hydrochloric acid and ammonia. For avoiding foam development, anti‐foamer was automatically added. The fed‐batch mode was indicated by the sharp loss of DO and commenced by feeding 17 L M9 supplementary medium at the speed of 100 mL/min. At the checkpoint of 6 h, 0.1 mM IPTG was added into the culture media at 28℃. The fermentation was stopped at 10 h and the cells were harvested by contrifugation (8000 rpm, 30 min). The time‐course parameters of fermentation were automatically measured and the samples of fermentation broth (10 mL) were taken out every 1 h. The values of OD_600_ were detected by microplate reader. The wet cell weight (WCW) of Peptibody strains were measured after contrifugation (8000 rpm, 10 min). The expression level was analyzed by SDS‐PAGE and the gel images were scanned to quantify the targeted protein via Bio‐rad Image System.

### 2.6 Scaling up Peptibody purification from the soluble fraction

The fermentation product was resuspended in 20 mM phosphate buffer (PB) supplemented with EDTA‐free protease inhibitor (Beyotime Biotechnology, Jiangsu, China) and lysed by homogenizer. The supernatant of cell lysate was subject to the purification procedures operated in ÄKTA™ Explorer 100 and the chromatographic media were Capto™S ImpAct, Phenyl Sepharose HP and rProtein A (GE Healthcare, Piscataway, NJ, USA). The supernatant was loaded into the pre‐equilibrated cation‐exchange column of 15.7 mL and isolated by gradient NaCl‐PB (0.1–1.0 M). Then, the procedure was amplified in a large‐scale column of 465.1 mL and isolated by 0.5, 0.7, 1.0 M NaCl‐PB. The fractions of 0.7 and 1.0 M NaCl‐PB were pooled together in the binding buffer containing 2 M NaCl‐PB and loaded into the pre‐equilibrated hydrophobic column of 42.4 mL. The bound protein was isolated by gradient saline solution from high to low (1.5 M, 1.0 M, 0.5 M NaCl‐PB, 20 mM PB and H_2_O). The fractions from hydrophobic chromatography were pooled together in the binding buffer 20 mM PB and loaded into the pre‐equilibrated Protein A column of 5 mL. The bound protein was isolated by 10 mM NaCl‐PB and 20 mM citrate buffer‐PB (CB‐PB, pH 3.6). All the procedures were performed at 4℃ and the pH of buffers except 20 mM CB‐PB were 7.4. The time‐course parameters of purification were automatically measured by ÄKTA™ Explorer 100, including protein ultraviolet absorbance (UV), Cond, buffer concentration (Conc), Pressure and pH. Samples were collected in response to the absorbance value and analyzed by SDS‐PAGE assay. The gel images were scanned to quantify the targeted protein and calculate the recovery via Bio‐rad Image System. The purified product was concentrated in 0.015 M PBS by ultra concentrators. The purity and the HCP, DNA and endotoxin residues were detected by HPLC, ds‐ELISA, real‐time quantitative PCR (qRT‐PCR), fluorescence staining and limulus amebocyte lysate (LAL) assays, respectively.

### 2.7 HPLC

Following the supplier's protocol, Agilent 1260 Infinity system equipped with auto‐sampler, binary pump, thermostat, diode array detector and chemstation software and HPLC column Ultimate XB‐C4 (4.6 × 250 mm, 5 µm, Welch, USA) was used for purity analysis. The temperature of column was 30℃ and the mobile phase A/B was H_2_O/methyl alcohol, followed by the elution procedures: 95% A/5% B in 0 min, 90% A/10% B in 5 min and 50% A/50% B in 10 min. The purity was calculated by dividing the peak area of Peptibody and other protein.

### 2.8 ELISA

Every 2 wk, BALB/c mice were subcutaneously immunized with Peptibody vaccine (100 µg/mouse) emulsified completely with an equal volume of Freund's adjuvant (Sigma, St Louis, MO, USA) and PBS of same volume for three times. Ten days after the last immunization, blood samples were extracted and the titers of anti‐Peptibody antibody were detected by indirect ELISA. The recombinant human bFGF and VEGFA (R&D Systems, Minneapolis, MN, USA) were coated into the 96‐well plates at the concentration of 50 ng/well and the plates were incubated overnight at 4℃. After 1‐h blocking treatment of 5% non‐fat milk, the plates were incubated with a serial dilution of sera from Peptibody and PBS‐vaccinated mice, followed by 45‐min HRP‐conjugated goat anti‐mouse IgG (1:2000, Sangon Biotech, Shanghai, China) incubation at 37℃. The HCP of purified product was detected by double‐antibody sandwich ELISA (ds‐ELISA) using *E. coli* HCP ELISA kit (Cygnus, Beijing, China). The absorbance of 450 nm was read by a microplate reader. The antibody titers were defined as the reciprocal of serum dilution and the HCP residue was calculated according to standard curve.

### 2.9 Real‐time quantitative PCR

Residual DNA of purified product was extracted and amplified with primers (23S‐F: 5′‐TGGAAA GGC GCG CGA‐3′, 23S‐R: 5′‐GTG TCC CGC CCT ACTCA‐3′) and SYBR Green PCR Mix (TAKARA, Shiga, Japan) using 7500 Fast Real‐Time PCR System (Applied Biosystems, Foster City, CA, USA). The amplification curves, melt curves, and cycle threshold were analyzed by ABI 7500 FAST software. Total DNA of Peptibody strains served as the control.

### 2.10 Tumor transplantation in mice and immunohistochemistry (IHC) assay

At a 2‐wk interval, the C57BL/6 mice (n = 4/group) were immunized with Peptibody vaccine (100 µg/mouse) emulsified completely with an equal volume of Freund's adjuvant for three times. PBS (n = 4/group) served as the negative control. Ten days after the last immunization, LL‐2 cells (1 × 10^6^/mouse) were injected into the left shoulder flank. The tumor growth was measured every 3 days and the tumor volume was calculated by the following formula: V (mm^3^) = π/6(a × b^2^). a = length, b = width. The tumor tissues were weighed and imaged after the mice were sacrificed. Tumor tissues from the mouse model were fixed in 4% paraformaldehyde and embedded in paraffin. After deparaffinization, the samples were cut into sections of 5 mm. The endogenous peroxidase activity was repressed and the sections were blocked by 5% BSA and 0.05% Triton X‐100. Primary antibodies used were rabbit anti‐mouse CD31 (1:300, Abcam, Cambridge, MA, USA) and anti‐mouse LYVE‐1 (1:1500, Abcam) (37℃, 1 h). Secondary antibody used was HRP‐conjugated goat anti‐rabbit IgG (1:200, Servicebio, Wuhan, China) (37℃, 45 min). The sections were stained with 3,3′‐diaminobenzidine and hematoxylin and visualized by a bright‐field microscope (Olympus, Tokyo, Japan). Positive reactions were defined as those showing brown signals. The CD31 and LYVE‐1 positive cells were counted in five random fields. The tumor tissue of the PBS‐vaccinated mice served as the negative control.

### 2.11 Cell viability and migration

LL‐2 cells (1 × 10^3^/well) were transferred to 96‐well plates and activated by 10 ng/mL bFGF. The cells were treated with anti‐Peptibody antibody at the concentration from 12.5 to 400 µg/mL for 72 h and CCK‐8 reagent (Dojindo, Kumamoto, Japan) of 10 µL was added in the dark. The absorbance at 450 nm was read by a microplate reader. LL‐2 cells (3 × 10^5^/well) suspended in serum‐free medium were transferred to the upper transwell chambers coated with Matrigel (BD Biosciences, San Jose, CA, USA) and incubated with 200 µg/mL anti‐Peptibody antibody. Medium containing 10% FBS was transferred to the bottom chambers. After 24 h, the stained cells were imaged using an inverted microscope (Olympus, Tokyo, Japan) and the migrated cells were counted in five random fields.

### 2.12 Western blotting and antibodies

LL‐2 cells (3 × 10^5^/well) were transferred to the 6‐well plates and activated by 10 ng/mL bFGF. The cells were treated with anti‐Peptibody antibody at the concentration from 25 to 400 µg/mL. After 1 h, the cells were lysed by RIPA buffer containing protease and phosphatase inhibitors [24]. The proteins of cell lysate (20 µg) were separated by SDS‐PAGE and transferred onto PVDF membranes (Millipore, Bedford, MA, USA), followed by blocking treatment of 5% non‐fat milk. Primary antibodies used were rabbit anti‐mouse VEGFA (1:10,000, Abcam), anti‐t/p‐Akt (1:1000, 1:2000), anti‐t/p‐MAPK (1:1000, 1:2000) and anti‐GAPDH (1:1000, Cell Signaling Technology, Beverly, MA, USA) (37℃, 1 h). Secondary antibody used was HRP‐conjugated goat anti‐rabbit IgG (1:5000, Servicebio) (37℃, 45 min). The protein brands were detected using ECL kit (Millipore) and the signal intensities were calculated by ImageJ software. GAPDH served as the loading control.

### 2.13 Statistical analysis

The experiments were individually repeated three times and the data were shown as mean ± SD. The statistical significance between groups was detected by ANOVA test and SPSS 19.0 software. *p* < 0.05 was defined as statistically significant.

## 3 RESULTS

### 3.1 Biological characterizations of Peptibody strains

**FIGURE 1:**
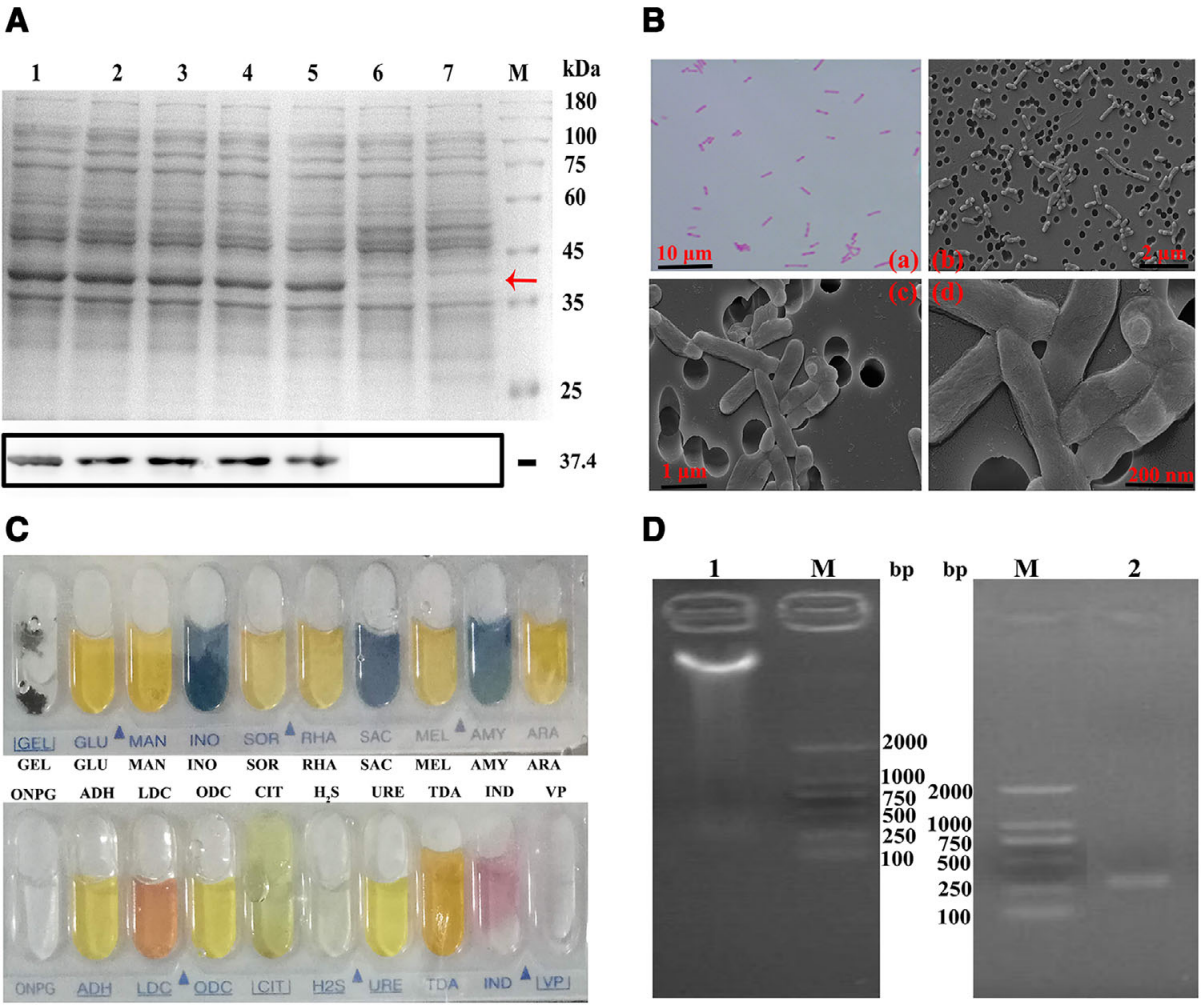
The biological characterizations of Peptibody strains. (A) The expression levels of Peptibody strains were analyzed by SDS‐PAGE and Western blotting assays. Lanes 1–5, cell lysate at the storage of 1, 3, 6, 9 and 12 months; Lane 6, cell lysate before induction; Lane 7, cell lysate of the empty vector; Lane M, protein molecular weight marker. The target protein was 37.4 kDa in which the red arrow pointed at. (B) The morphology of Peptibody strains was detected by Gram staining and scanning electron microscope assays. Scale bars: (a) 10 µm; (b) 2 µm; (c) 1 µm; (d) 200 nm. (C) The biochemical properties of Peptibody strains were detected by API 20E assay. (D) The species identification of Peptibody strains was conducted by 16S rDNA sequencing. Lane 1, the total bacterial DNA; Lane 2, PCR product of 16S rDNA V4 region; Lane M, nucleic acid marker

The synthesized genetic linkage of bFGF/VEGFA‐Fc was inserted into the expression vector pET‐28a and transformed to *E. coli* strain of BL21. Before fermentation, a library of Peptibody strains that stably expressing recombinant protein was needed. The biological characterizations of Peptibody strains were detected by storage, Gram staining, SEM, API 20E and 16S rDNA sequencing assays. Consequently, the target protein was stably expressed after a long‐time storage, at a level exceeding 20% of total cell protein (Figure 1A). In Gram staining, the result was red and the bacteria scattered as shot rods. In the ultrastructure, no spore, capsule, chlamydia, mycoplasma, phage, virus and other pathogens were observed (Figure 1B). API 20E is a standard identification system for *E. coli* and Gram‐negative rods using miniaturized biochemical reactions and a species database [25]. The identified results were showed in Figure 1C, in accordance with the biochemical properties of *E. coli*. Importantly, 16S rDNA of high conservation was sequenced and compared to NCBI, with a matching to *E. coli* over 98% (Figure 1D). Thus, the species of Peptibody strains was identified as *E. coli* and a stable library was successfully established. The best‐performing strains were selected for following fermentation.

### 3.2 Optimization of Peptibody production from flask to 10‐L bioreactor

**FIGURE 2:**
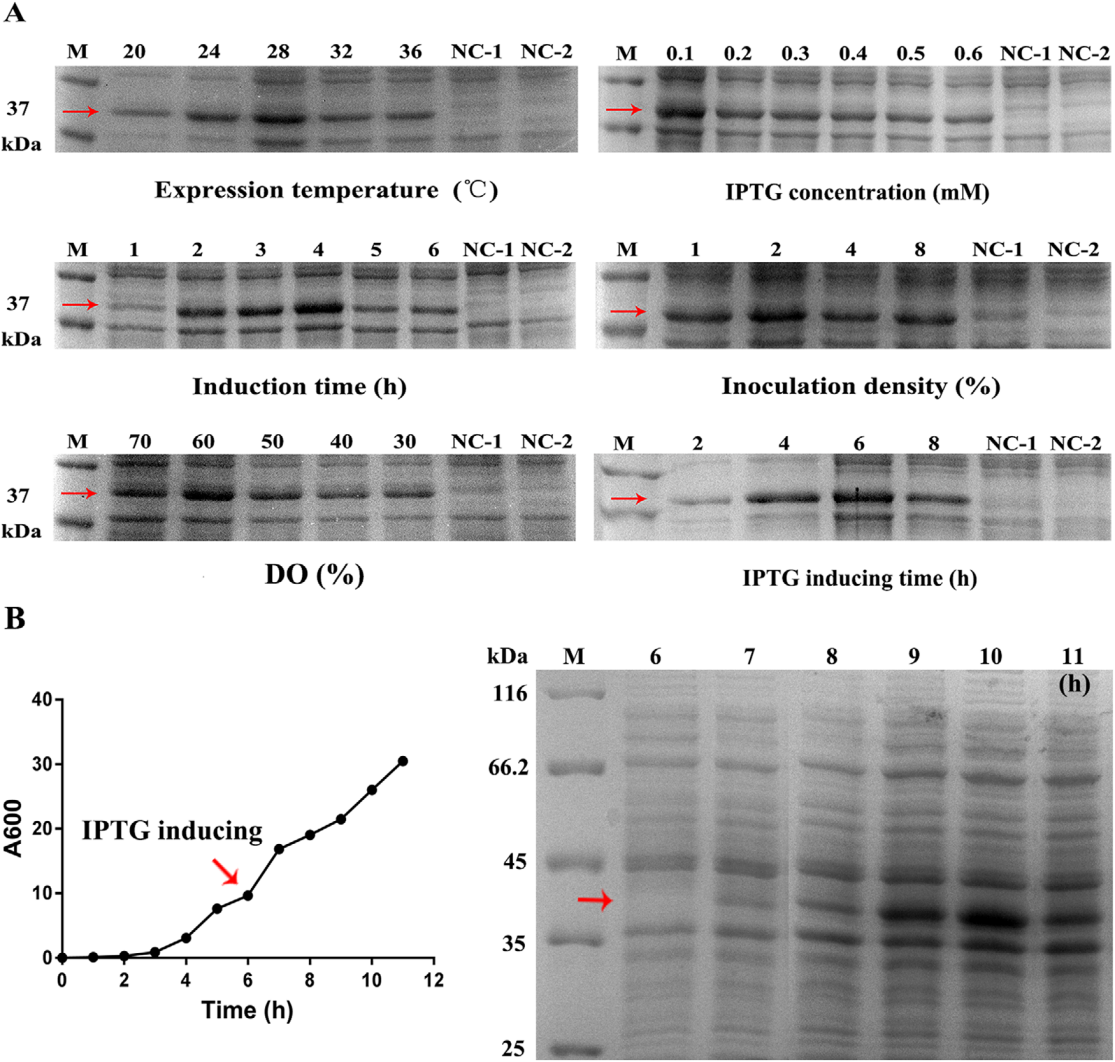
Optimization of Peptibody production from flask to 10‐L bioreactor fermentation. (A) SDS‐PAGE analysis of flask fermentation optimization. The Peptibody strains were cultured and induced in different conditions. Lane M, protein molecular weight marker; Lane NC‐1, cell lysate before induction; Lane NC‐2, cell lysate of the empty vector. For expression temperature, lanes from left to right, cell lysate at the temperature of 20, 24, 28, 32, and 36℃; For induction concentration, lanes from left to right, cell lysate at the concentration of 0.1, 0.2, 0.3, 0.4, 0.5, and 0.6 mM IPTG; For induction time, lanes from left to right, cell lysate at the induction of 1, 2, 3, 4, 5 and 6 h; For inoculation density, lanes from left to right, cell lysate at the density of 1, 2, 4, and 8%; For DO, lanes from left to right, cell lysate at the dissolved oxygen (DO) above 30, 40, 50, 60 and 70%; For inducer added time, lanes from left to right, cell lysate at the timing of 2, 4, 6, and 8 h. (B) The growth curve and SDS‐PAGE analysis of Peptibody production in 10‐L bioreactor. The fermentation mode were controlled at 37℃, pH 7.0 ± 0.2 and DO 30%. The inoculation density was 2% and the feeding speed was set at 10.0 mL/min. Lane M, protein molecular weight marker; Lanes from left to right, IPTG induction from 6 to 11 h. The target protein was 37.4 kDa in which the red arrow pointed at

Before large‐scale Peptibody production, the culture conditions and fermentation mode were optimized from flask to 10‐L bioreactor. The Peptibody strains were cultivated in the 500 mL flasks containing 200 mL M9 growth medium for expression condition optimization, using IPTG as an inducer. In Figure 2A, the culture conditions optimized included inoculation density, DO, induction temperature, IPTG concentration, IPTG inducing time and induction time. The best conditions in the shake flasks were 2%, over 30%, 28℃, 0.1 mM, 6 and 4 h, with highest expression level of target protein.

To achieve a high cell‐density culture, the fermentation mode from the flask was then optimized in 10‐L bioreactor. The growing temperature was 37℃ and DO was maintained over 30%. The activated seed were inoculated at a density of 2% and exhibited a 2‐h lag growth phase in which Peptibody was accumulated at a low level. Following the supplementary procedure at 10 mL/min, cell growth then entered into an exponential phase. IPTG of 0.1 mM was added at 6 h, 28℃, the middle exponential phase, and Peptibody started to increase rapidly up to 10 h. The fermentation should be stopped at this moment because the target protein was no longer increased. The time‐course profiles of cell growth and analysis of Peptibody expression were presented in Figure 2B, with a final WCW of 92.75 g/L and a expression level exceeding 20% of total cell protein. Thus, the fermentation mode from the flask was applicable to 10‐L bioreactor.

### 3.3 Large‐scale Peptibody production in 100‐L bioreactor

**FIGURE 3:**
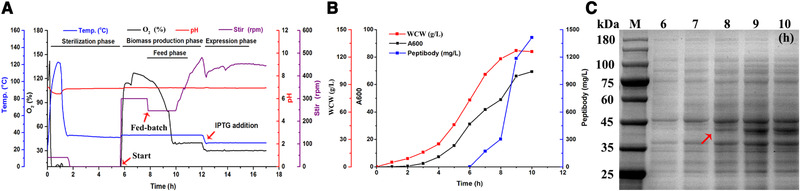
Large‐scale fermentation of Peptibody production in 100‐L bioreactor. (A) The time‐course profile of Peptibody production in 100‐L bioreactor. The fermentation mode was controlled at 37℃, pH 7.0 ± 0.2 and dissolved oxygen (DO) 30%. The inoculation density was 2% and the feeding speed was set at 100.0 mL/min. The denoted signals were temperature (blue), DO (black), pH (red) and stirr (purple) and the denoted phases were biomass production, feed and expression. (B) The growth and expression curves of Peptibody strains during fermentation. (C) SDS‐PAGE analysis of Peptibody production during fermentation. Lane M, protein molecular weight marker; Lanes from left to right, IPTG induction from 6 to 10 h. The bands corresponding to the target protein were indicated by red arrows, 37.4 kDa

On the basis of 10‐L process, we scaled up the Peptibody production in 100‐L bioreactor. The growing temperature, DO level and inoculation density were the same as Section 3.2. Following the supplementary procedure at 100 mL/min, the growth speed got significantly improved from 2 h. IPTG of 0.1 mM was added at 6 h, 28℃ and Peptibody started to increase rapidly. The fermentation was also stopped at 10 h. The time‐course profiles of cell growth and expression analysis were presented in Figure 3A–C, with WCW 126 g/L, production 1.41 g/L and productivity 0.35 g/(L∙h). Thus, the fermentation mode from 10‐L bioreactor was also applicable to 10‐L bioreactor. From flask to 100‐L bioreactor, we successfully produced Peptibody in a large‐scale fed‐batch fermentation, providing an avenue for economical and industrial Peptibody production.

### 3.4 Large‐scale Peptibody purification and the purity analysis

**FIGURE 4:**
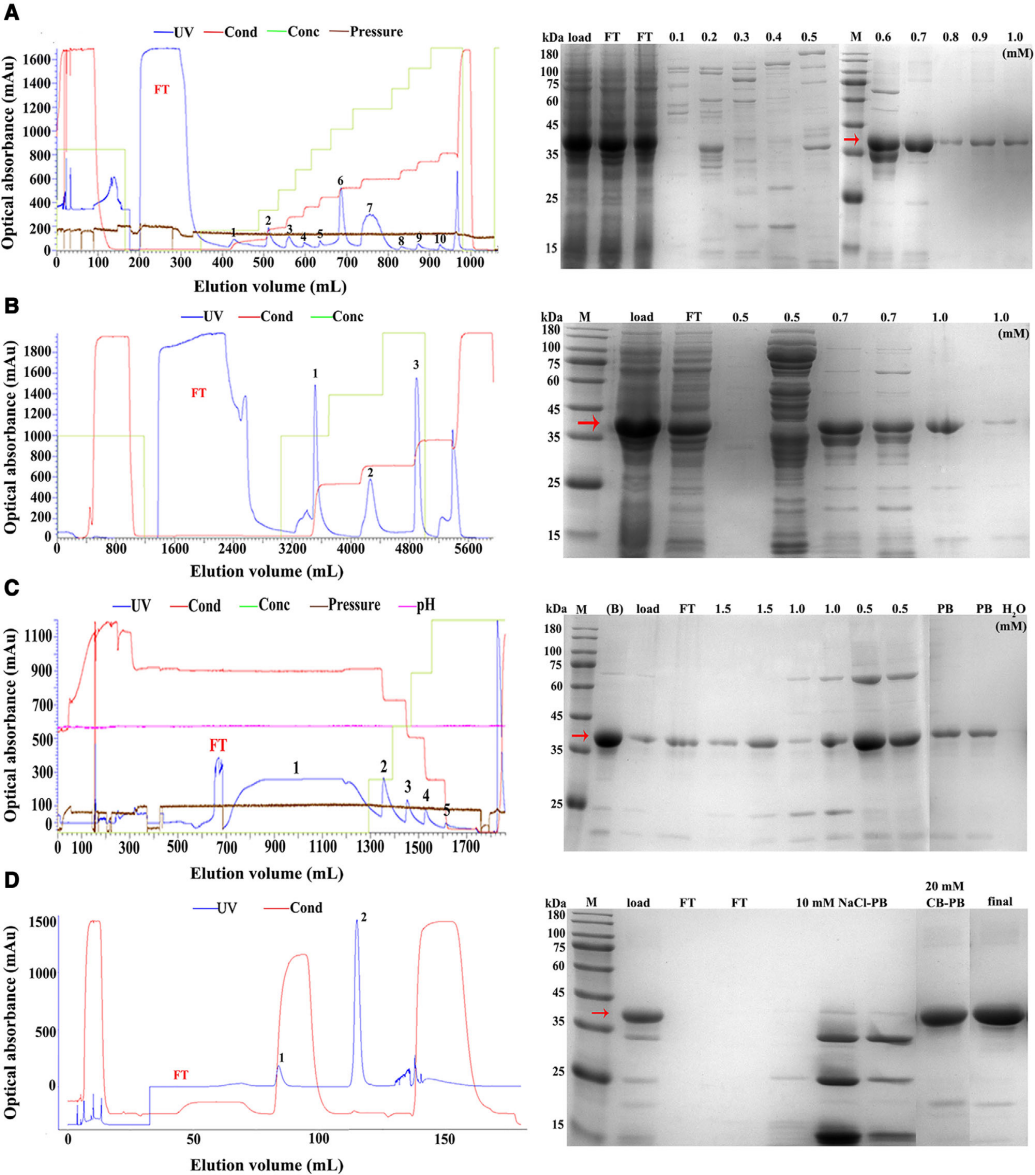
Large‐scale purification of Peptibody fusion protein. (A) The purification procedures and SDS‐PAGE analysis of 15.7 mL cation‐exchange chromatography. The supernatant of cell lysate was mixed with PB balance buffer and loaded into the cation‐exchange column. Peaks 1–10, the gradients of NaCl‐PB from 0.1 to 1.0 M. Lanes from left to right, supernatant of cell lysate (load), flow through (FT), elution of 0.1 to 1.0 M NaCl‐PB. (B) The purification procedures and SDS‐PAGE analysis of 465.1 mL cation‐exchange chromatography. Peaks 1–3, 0.5, 0.7, and 1.0 M NaCl‐PB. Lanes from left to right, load, FT and elution of 0.5, 0.7, 1.0 M NaCl‐PB. (C) The purification procedures and SDS‐PAGE analysis of hydrophobic chromatography. The eluted samples of (B) were mixed with 2 M NaCl‐PB balance buffer and loaded into the hydrophobic column. Peaks 1–5, 1.5, 1, 0.5, 0 M NaCl‐PB and H_2_O. Lanes from left to right, the eluted sample of (B), the eluted sample of (B) with 2 M NaCl‐PB (load), FT, 1.5 M NaCl‐PB, 1.0 M NaCl‐PB, 0.5 M NaCl‐PB, 20 mM PB, H_2_O. (D) The purification procedures and SDS‐PAGE analysis of Protein A affinity chromatography. The eluted samples of (C) were mixed with 20 mM PB balance buffer and loaded into the Protein A column. Peaks 1–2, 10 mM NaCl‐PB and 20 mM CB‐PB. Lanes from left to right, the eluted sample of (C) with 20 mM PB (load), 10 mM NaCl‐PB, 20 mM CB‐PB, the final product concentrated in 0.015 M PBS (final). Lane M, protein molecular weight marker. The lines in blue, red, green, coffee, and purple were UV, condition, concentration, pressure, and pH in the columns, respectively. The bands corresponding to the target protein were indicated by red arrows, 37.4 kDa

The Fc domain was assembled to facilitate the downstream purification using Protein A affinity chromatography. The excessive HCP would inhibit the combination between Peptibody and Protein A column. Cation‐exchange was an alternative chromatography for Peptibody capture in the bacterial lysate with a large amount of HCP. In the small column of 15.7 mL, following the standard purification protocol, gradient washing steps with 0.1–1.0 M NaCl‐PB were conducted and the target protein was not isolated until the elution concentration arrived at 0.6 M (Figure 4A). Then, we amplified the capture procedure in the large‐scale column of 465.1 mL to confirm that 0.5 M NaCl‐PB could separate most HCP and the target protein was eluted in 0.7 and 1.0 M NaCl‐PB (Figure 4B). Thus, it was possible to capture Peptibody using cation‐exchange chromatography, with a high recovery of 82.66%. Next, hydrophobic chromatography was used to further reduce the HCP. The fractions of 0.7 and 1.0 M NaCl‐PB above were pooled together in 2 M NaCl‐PB and the target protein was isolated by NaCl‐PB at the concentration from high to low, with a recovery of 86.52% (Figure 4C). After that, the eluted fractions of hydrophobic chromatography were loaded to Protein A. The residual HCP was removed by 10 mM NaCl‐PB and the target protein was isolated by 20 mM CB‐PB, with a recovery of 90.61%. The purified product was pooled together and concentrated with 0.015 M PBS. The purity of final product was more than 90% and the total recovery was 60.28% by SDS‐PAGE assay (Figure 4D). Thus, the purification procedures of cation‐exchange, hydrophobic and Protein A chromatography allowed to obtain highly pure Peptibody in a large scale.

**FIGURE 5:**
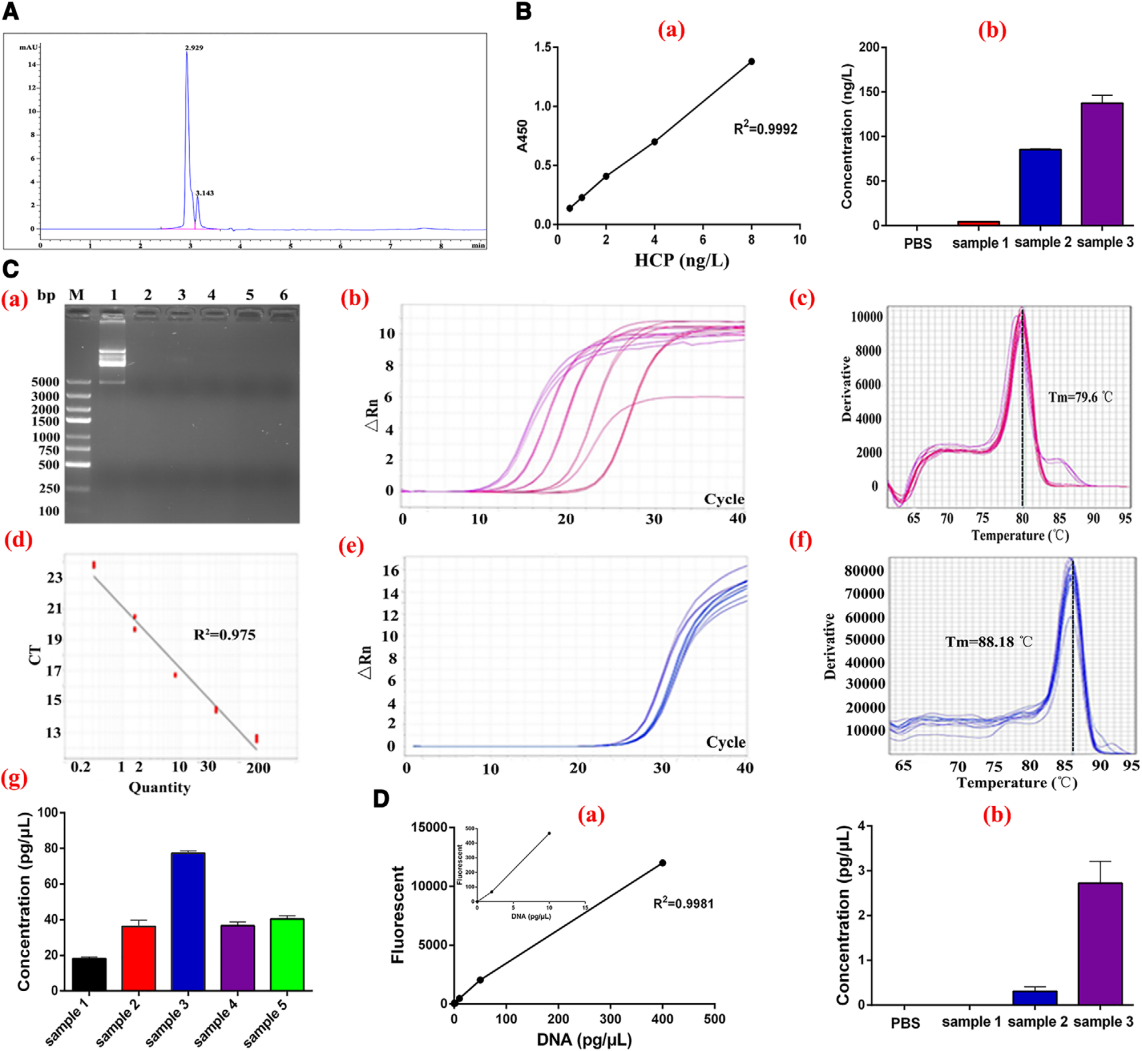
Purity analysis and the host cells protein (HCP), DNA and endotoxin residues. (A) HPLC analysis of the purified Peptibody. The purified samples were subjected to Ultimate XB‐C4 column and analyzed by Agilent 1260 Infinity system. (B) The residual HCP was detected by ds‐ELISA assay. (a) Standard curve. (b) Detection of three replicates. (C) The residual DNA was detected by qRT‐PCR assay. (a) Total DNA extraction. Lane M, nucleic acid marker. Lane 1, host bacteria; Lanes 2–6, five sample replicates. (b–d) The amplification, melt and standard curves of host DNA. (e, f) The amplification and melt curves of five sample replicates. (g) DNA concentration of five sample replicates. (D) The residual DNA was detected by fluorescent staining assay. (a) Standard curve. (b) Detection of three replicates

For biological agents from gene engineering, majority of fundings will sink into failures if safety findings occur [26]. Thereby, the purity and residues which produce side effects need further detection. The purified product was analyzed by HPLC and the retention time was 2.93 min, with a purity of 86.71% (Figure 5A). The HCP was detected by ds‐ELISA and the level was 75.88 ng/L (Figure 5B). We examined the DNA level by qRT‐PCR and fluorescence staining and the results showed that the DNA residuals were 1.01–41.80 pg/µl (Figure 5C,D). The endotoxin was less than 0.1 EU/µg, detected by LAL kit. The Peptibody vaccine was for human use and the HCP, DNA and endotoxin residues of the purified product were within the threshold, which indicated that the pernicious components could be totally purged in the purification procedures above.

### 3.5 Peptibody vaccine suppressed tumor growth and angiogenesis in vivo

**FIGURE 6:**
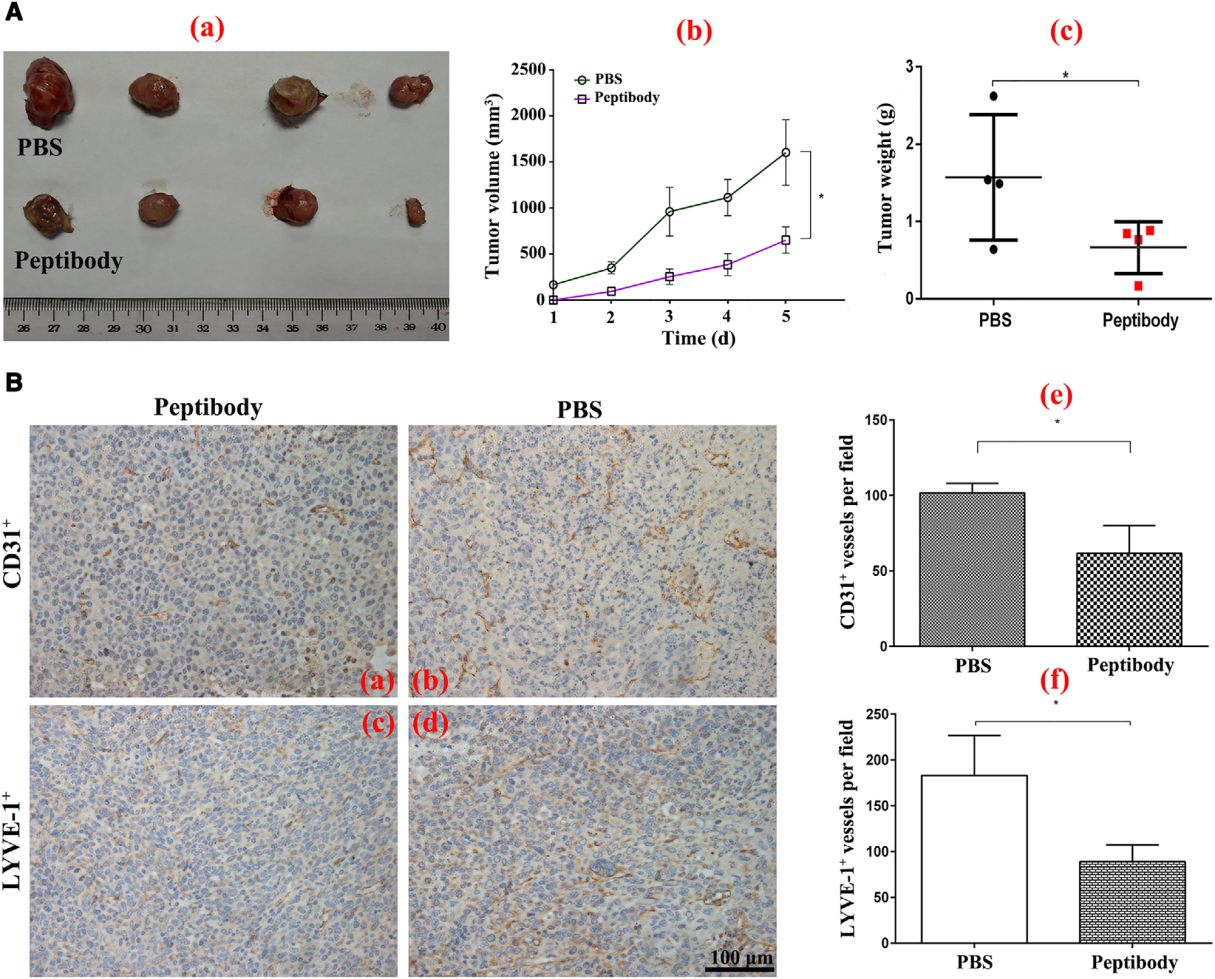
Inhibitory effects of Peptibody vaccine on tumor growth and angiogenesis in mice. (A) The C57BL/6 mice (n = 4/group) were subcutaneously immunized with PBS and Peptibody vaccine (100 µg/mouse). Ten days after the last vaccination, the mice were inoculated with LL‐2 cells (1 × 10^6^/mouse) and the tumor volume was measured every 3 days. (a) Tumor images. (b) Tumor growth curves. (c) Tumor weight. (B) Representative images and quantitation of microvessels and lymph vessels by immunohistochemistry assay. (a, b, e), tumor section and the quantitation of microvessels. (c, d, f), tumor section and the quantitation of lymph vessels. Scale bars, 100 µm. Data were shown as mean ± SD of three independent experiments (*p < 0.05 for the Peptibody‐vaccinated mice vs. the negative control. *p* Values were analyzed by two‐way ANOVA test, using SPSS 19.0 software)

Angiogenic proceed is abundant in lung cancer and the LL‐2 cells with high expression levels of bFGF and VEGFA were selected to investigate the tumor inhibition of Peptibody vaccine in vivo [27]. The C57BL/6 mice were immunized with Peptibody vaccine for three times and the LL‐2 cells were injected into the left shoulder flank 10 days after the last immunization. The tumor growth of Peptibody‐vaccinated mice was significantly inhibited in terms of both tumor volume and weight, with inhibition by 57.73% relative to the control (Figure 6A). The formation of microvessels and lymph vessels were analyzed by IHC assay. As shown in Figure 6B, the microvessels and lymph vessels were stained brown in the tumor sections they were significantly decreased by 39.34 and 51.45%. Therefore, Peptibody vaccine showed a dramatic protection to inhibit tumor growth and angiogenesis in mouse model, which might be used as a tumor therapeutic vaccine in the future.

**FIGURE 7:**
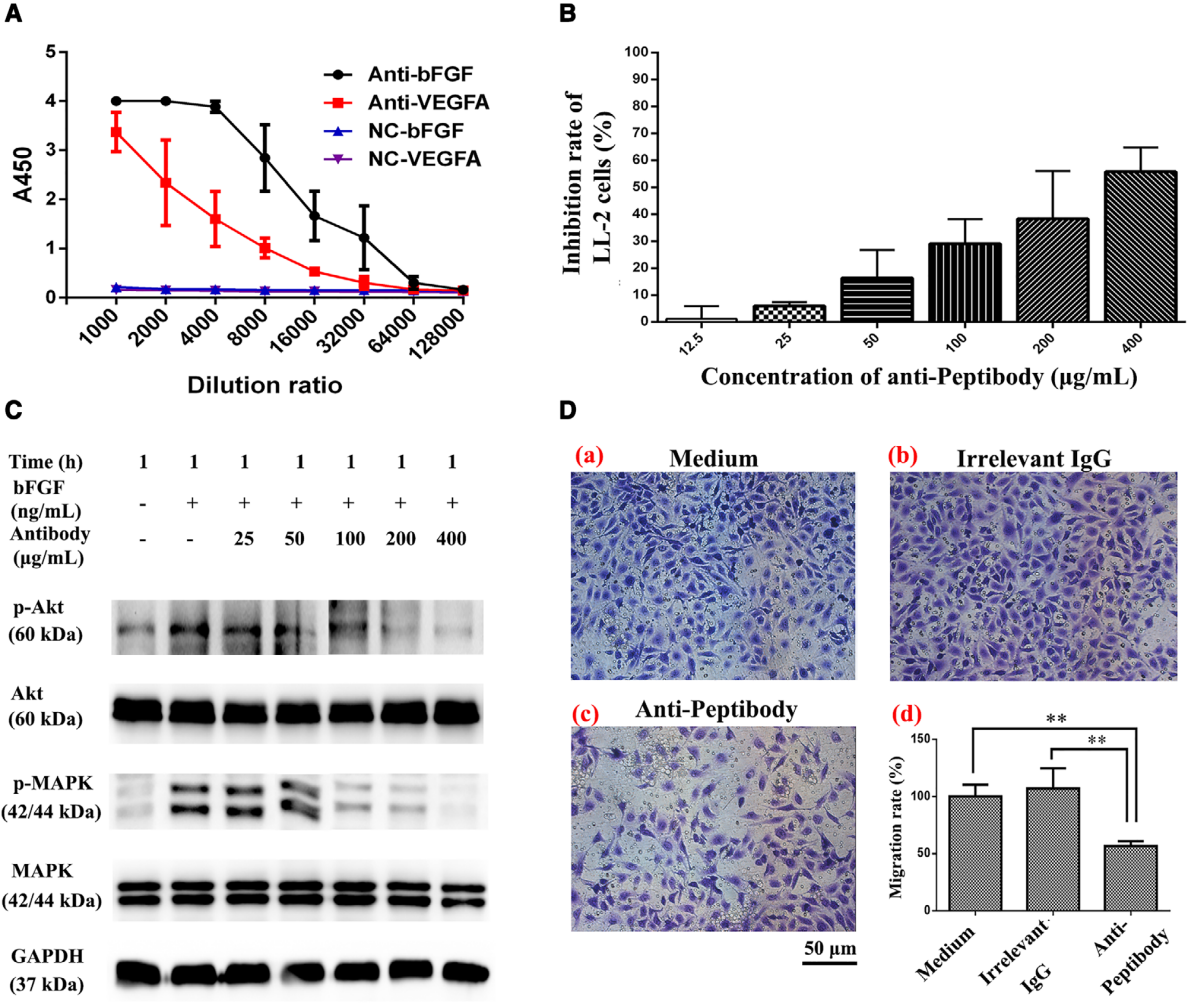
Inhibitory effects of anti‐Peptibody antibody on the proliferation, migration and Akt/MAPK signal pathways of LL‐2 cells. (A) Immunogenicity of the Peptibody vaccine in mice. The BALB/c mice were immunized with Peptibody vaccine and PBS for three times, respectively. Ten days after the last vaccination, the blood samples were collected. The anti‐Peptibody antibody was extracted and the titers were detected by ELISA assay. (B) The proliferation inhibition assay of LL‐2 cells. The cells (1 × 10^3^/well) were treated with anti‐Peptibody antibody at the concentration from 12.5 to 400 µg/mL. (C) Western blotting analysis of the phosphorylation of Akt and MAPK in LL‐2 cells. The cells (3 × 10^5^/well) were activated with bFGF and treated with anti‐Peptibody antibody at the concentration from 25 to 400 µg/mL. GAPDH served as the loading control. (D) Transwell chamber migration assay of LL‐2 cells. The cells (3 × 10^5^/well) were incubated with 200 µg/mL anti‐Peptibody antibody in the upper chamber for 24 h. PBS and irrelevant IgG served as the negative control. Scale bars, 50 µm. Data were shown as mean ± SD of three independent experiments (**p < 0.01 for the anti‐Peptibody antibody vs. the control. *p* Values were analyzed by one‐way ANOVA test, using SPSS 19.0 software)

To investigate the molecular mechanism about the inhibitory effects above, we further evaluated the immunogenicity of Peptibody vaccine. In BALB/c mice, it could elicit high‐titer anti‐bFGF (1:32,000) and anti‐VEGFA (1:8000) antibodies. There was no specific immunogenicity to negative control group (Figure 7A).

### 3.6 Anti‐Peptibody antibody inhibited the proliferation and migration of LL‐2 cells by decreasing the Akt/MAPK signal pathways

Assays of CCK‐8, signal pathway and transwell chamber were conducted to investigate the inhibitory effects of anti‐Peptibody antibody on tumor cell proliferation and migration. The bFGF activated LL‐2 cells were treated with serially diluted anti‐Peptibody antibody (12.5–400 µg/mL) and the proliferation was suppressed in a dose‐manner, with inhibition by 57.87 ± 7.13% at 400 µg/mL (Figure 7B). Moreover, the phosphorylation of Akt and MAPK were reduced in a dose‐manner (Figure 7C) and the migration of LL‐2 cells treated with 200 µg/mL anti‐Peptibody antibody was significantly repressed, with migration rate of 56.55 ± 4.20% (Figure 7D). The results indicated that anti‐Peptibody antibody could inhibit the proliferation and migration of LL‐2 cells by decreasing the Akt/MAPK signal pathways.

## 4 DISCUSSION

Inhibition of tumor angiogenesis has been become a promising strategy for cancer therapy but resistance to single pathway has been a great concern [28]. For enduring clinical responses, it necessitates multiple targetable factors for developing novel tumor angiogenesis inhibitor. We applied Fc fusion concept to bFGF/VEGFA pathways and the recombinant Peptibody contained antigenic epitopes derived from these two factors. In mouse model, the tumor growth and angiogenesis were suppressed by Peptibody vaccine, which could elicit superior immune responses of anti‐bFGF and anti‐VEGFA antibodies. In vitro experiments, the anti‐Peptibody antibody could inhibit the proliferation and migration of tumor cells by decreasing the Akt/MAPK signal pathways. Although the inhibitory effects of Peptibody vaccine have been shown to partly inhibit tumorigenesis and tumor angiogenesis, molecular mechanisms involved are still largely elusive, particularly how Peptibody targets and regulates angiogenesis networks and related downstream genes. We assumed that Peptibody vaccine with dramatic immunogenicity could neutralize bFGF and VEGFA in tumor microenvironment via the anti‐bFGF and anti‐VEGFA antibodies it elicited. The activation of Akt/MAPK signal pathways were blockaded, resulting tumor progression and angiogenesis inhibition.

We established large‐scale production and purification of Peptibody vaccine to support the industrial production and pre‐clinical study in the future. The controlled promoter of prokaryotic expression system separated cell growth and Peptibody synthesis into two consecutive phases, allowing overexpression of the encoded genes in pET‐28a [29]. The bacterial proliferation and protein expression are complex and numerous factors such as strain property, plasmid stability, medium composition, culture conditions and induction mode need to be improved during fermentation [30]. We established a library of Peptibody strains with stable properties and we successfully produced Peptibody via fermentation from flask to 100‐L bioreactor. The suitable fermentation conditions were M9 medium, growth temperature 37℃, DO over 30%, pH 7.0 ± 0.05, inoculation density 2%, feed phase from 2 to 5 h, IPTG added time 6 h, IPTG concentration 0.1 mM, induction temperature 28℃ and induction time 4 h, which could achieve a high cell‐density biomass and a high proportion of soluble recombinant protein. The fermentation of Peptibody strains could be finished in a short time in IPTG induction, with considerable WCW, protein expression and productivity. The large‐scale process we developed provided an avenue for economical and industrial Peptibody production in the future.

In the purification procedures, cation‐exchange cloumn of Capto™S ImpAct is rather applicable for protein capture, conferring multiple superiorities of low cost, low toxicity, high capacity, high performance and fast flow [31]. The recombinant Peptibody (pI 8.93) carried about two units positive charges in 20 mM PB (pH 7.4), which made a strong combination to the groups with negative charges in this column. It needed high‐salinity buffer for protein isolation so that the binding kept stable before 0.6 M NaCl‐PB. The residual DNA and endogenous protein like endotoxin carrying negative charges in 20 mM PB were barried out in this column [32, 33]. The hydrophobic groups of Peptibody could be exposed in 2.0 M NaCl‐PB and hydrophobic column of Phenyl Sepharose HP was added to further clear away some endogenous protein with little hydrophobic groups [34]. With most HCP removed out, Peptibody was successfully combined with Protein A and purified in a high‐affinity manner. Importantly, the HCP, DNA and endotoxin residues were controlled at a very low level. Despite the considerable purity and recovery of each step, the total recovery was barely 60.28%. Therefore, pure product of Peptibody could be harvested from these purification procedures, demonstrating potential for industrial production, but the recovery of each step needed further improvement.

## 5 CONCLUSION

The tumor vaccine multi‐epitope Peptibody with bFGF/VEGFA we developed showed significant inhibitory effects on tumor progression and angiogenesis in vivo and vitro experiments, which might be used as a potential therapeutic vaccine for tumor treatment. The library of Peptibody strains with stable biological characterizations was constructed. For a large‐scale production and purification of Peptibody fusion protein, we had successfully established the upstream fermentation from flask to 100‐L bioreactor and downstream recovery of Peptibody from small to large‐scale purification, which helps its industrial production and pre‐clinical study for reference in the future.

## CONFLICT OF INTEREST

The authors have declared no conflict of interest.
