# Corrigendum

**DOI:** 10.1002/elsc.202270073

**Published:** 2022-08-03

**Large‐scale production, purification, and function of a tumor multi‐epitope vaccine: Peptibody with bFGF/VEGFA**

Ligang Zhang, Chengcheng Jiang, Xi Chen, Jiangtao Gu, Qifang Song, Hui Zhong, Sheng Xiong, Qingfeng Dong, Jin‐Chen Yu, Ning Deng

Eng. Life Sci. 2020, 20, 422–436

https://doi.org/10.1002/elsc.202000020

First published: 29 July 2020

For the above‐mentioned article, concerns about image integrity were raised by a third party. The authors apologize for their carelessness and make corrections to their published paper. Figures 1, 2, 4 and 7 are replaced. Original raw data and additional experiments were evaluated in detail by Wiley and the editors. The conclusions of the original manuscript remain unchanged.

**FIGURE 1:**
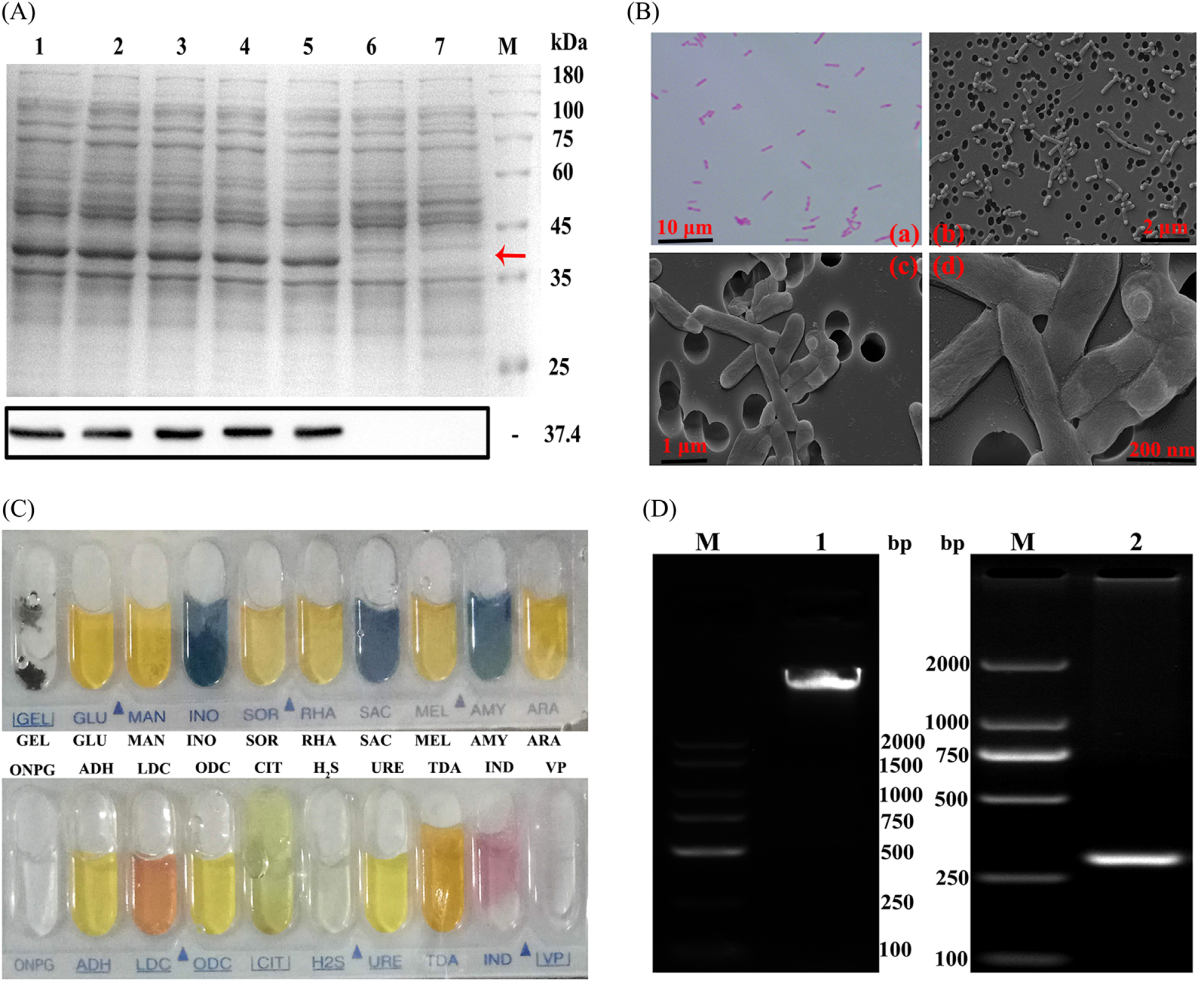
The biological characterizations of Peptibody strains. (A) The expression levels of Peptibody strains were analyzed by SDS‐PAGE and Western blotting assays. Lanes 1–5, cell lysate at the storage of 1, 3, 6, 9 and 12 months; Lane 6, cell lysate before induction; Lane 7, cell lysate of the empty vector; Lane M, protein molecular weight marker. The target protein was 37.4 kDa in which the red arrow pointed at. (B) The morphology of Peptibody strains was detected by Gram staining and scanning electron microscope assays. Scale bars: (a) 10 μm; (b) 2 μm; (c) 1 μm; (d) 200 nm. (C) The biochemical properties of Peptibody strainswere detected by API 20E assay. (D) The species identification of Peptibody strains was conducted by 16S rDNA sequencing. Lane 1, the total bacterial DNA; Lane 2, PCR product of 16S rDNA V4 region; Lane M, nucleic acid marker

**FIGURE 2:**
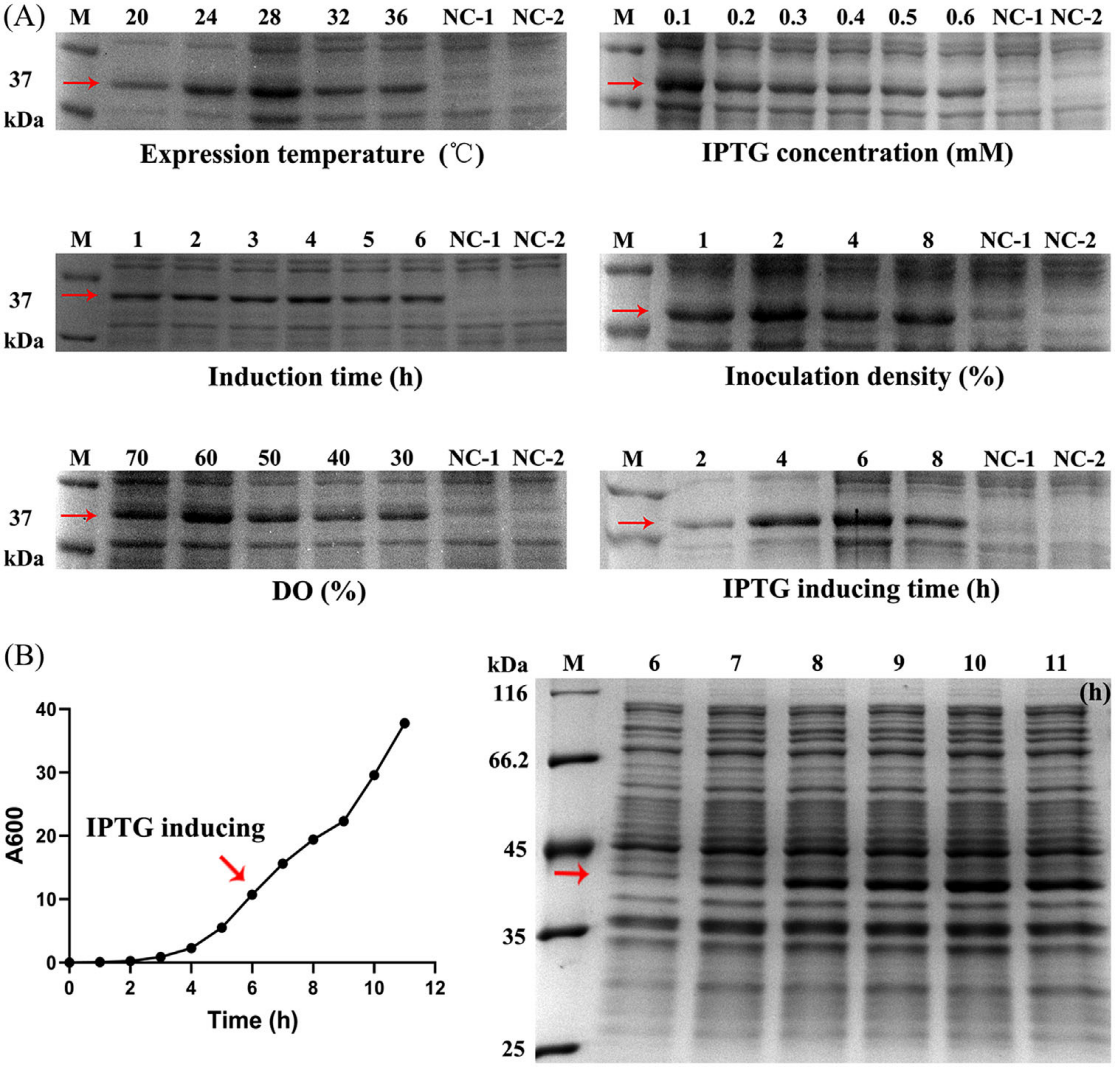
Optimization of Peptibody production from flask to 10‐L bioreactor fermentation. (A) SDS‐PAGE analysis of flask fermentation optimization. The Peptibody strains were cultured and induced in different conditions. Lane M, protein molecular weight marker; Lane NC‐1, cell lysate before induction; Lane NC‐2, cell lysate of the empty vector. For expression temperature, lanes from left to right, cell lysate at the temperature of 20, 24, 28, 32, and 36°C; For induction concentration, lanes from left to right, cell lysate at the concentration of 0.1, 0.2, 0.3, 0.4, 0.5, and 0.6 mM IPTG; For induction time, lanes from left to right, cell lysate at the induction of 1, 2, 3, 4, 5 and 6 h; For inoculation density, lanes from left to right, cell lysate at the density of 1, 2, 4, and 8%; For dissolved oxygen (DO), lanes from left to right, cell lysate at the DO above 30, 40, 50, 60 and 70%; For inducer added time, lanes from left to right, cell lysate at the timing of 2, 4, 6, and 8 h. (B) The growth curve and SDS‐PAGE analysis of Peptibody production in 10‐L bioreactor. The fermentation mode was controlled at 37°C, pH 7.0 ± 0.2 and DO 30%. The inoculation density was 2% and the feeding speed was set at 10.0 mL/min. Lane M, protein molecular weight marker; Lanes from left to right, IPTG induction from 6 to 11 h. The target protein was 37.4 kDa in which the red arrow pointed at

**FIGURE 4:**
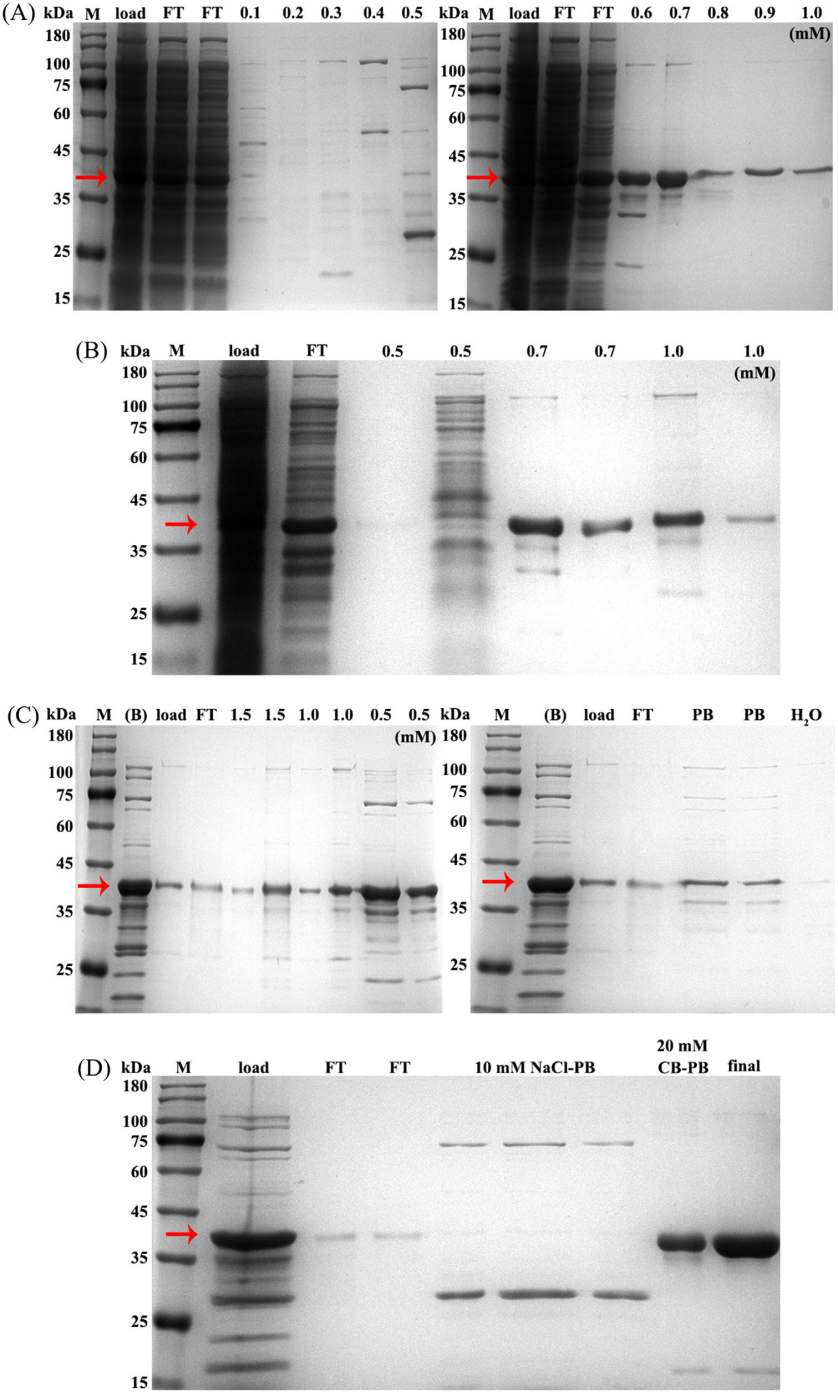
Large‐scale purification of Peptibody fusion protein. (A) The purification procedures and SDS‐PAGE analysis of 15.7 mL cation‐exchange chromatography. The supernatant of cell lysate was mixed with PB balance buffer and loaded into the cation‐exchange column. Lanes from left to right, supernatant of cell lysate (load), flow through (FT), elution of 0.1 to 1.0 M NaCl‐PB. (B) The purification procedures and SDS‐PAGE analysis of 465.1 mL cation‐exchange chromatography. Lanes from left to right, load, FT and elution of 0.5, 0.7, 1.0 M NaCl‐PB. (C) The purification procedures and SDS‐PAGE analysis of hydrophobic chromatography. The eluted samples of (B) were mixed with 2 M NaCl‐PB balance buffer and loaded into the hydrophobic column. Lanes from left to right, the eluted sample of (B), the eluted sample of (B) with 2 M NaCl‐PB (load), FT, 1.5 M NaCl‐PB, 1.0 M NaCl‐PB, 0.5 M NaCl‐PB, 20 mM PB, H_2_O. (D) The purification procedures and SDS‐PAGE analysis of Protein A affinity chromatography. The eluted samples of (C) were mixed with 20 mM PB balance buffer and loaded into the Protein A column. Lanes from left to right, the eluted sample of (C) with 20 mM PB (load), FT, 10 mM NaCl‐PB, 20 mM CB‐PB, the final product concentrated in 0.015 M PBS (final). Lane M, protein molecular weight marker. The bands corresponding to the target protein were indicated by red arrows, 37.4 kDa

**FIGURE 7:**
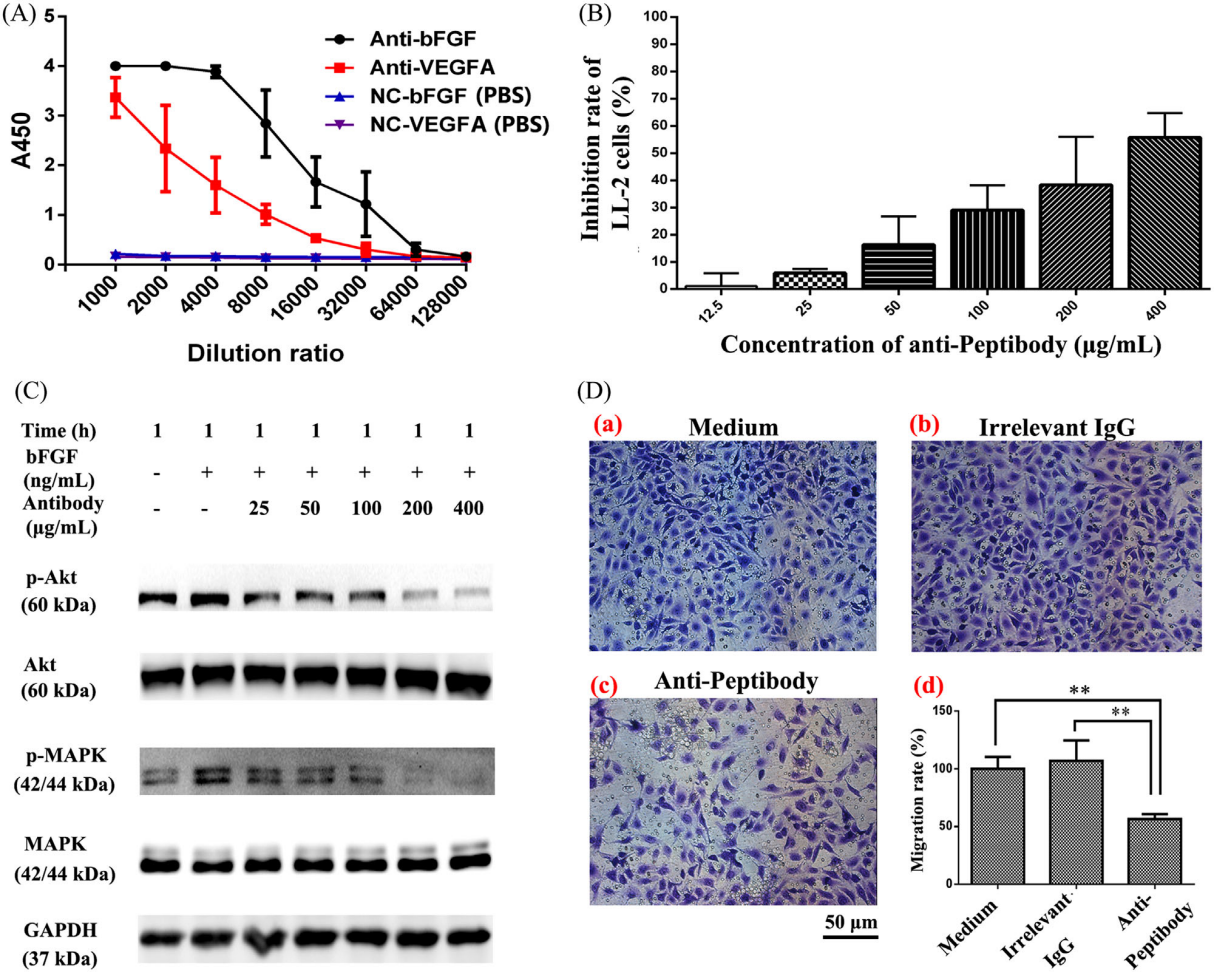
Inhibitory effects of anti‐Peptibody antibody on the proliferation, migration and Akt/MAPK signal pathways of LL‐2 cells. (A) Immunogenicity of the Peptibody vaccine in mice. The BALB/c mice were immunized with Peptibody vaccine and PBS for three times, respectively. Ten days after the last vaccination, the blood samples were collected. The anti‐Peptibody antibody was extracted and the titers were detected by ELISA assay. (B) The proliferation inhibition assay of LL‐2 cells. The cells (1 × 10^3^/well) were treated with anti‐Peptibody antibody at the concentration from 12.5 to 400 μg/mL. (C) Western blotting analysis of the phosphorylation of Akt and MAPK in LL‐2 cells. The cells (3 × 10^5^/well) were activated with bFGF and treated with anti‐Peptibody antibody at the concentration from 25 to 400 μg/mL. GAPDH served as the loading control. (D) Transwell chamber migration assay of LL‐2 cells. The cells (3 × 10^4^/well) were incubated with 200 μg/mL anti‐Peptibody antibody in the upper chamber for 24 h. PBS and irrelevant IgG served as the negative control. Scale bars, 50 μm. Data were shown as mean ± SD of three independent experiments (**p < 0.01 for the anti‐Peptibody antibody vs. the control. p Values were analyzed by one‐way ANOVA test, using SPSS 19.0 software)

**Figure** **1**: In Figure 1A the Western Blot bands had been cut off and a sliced image was shown. The authors explained this with additional lanes not being shown in the final figure. They repeated the Western Blot and results are given below. In Figure 1D the gel image appeared to consist of two merged lanes, the author explained this with an additional lane which does not belong to this manuscript between lane M and lane 2, the raw data was provided for evidence. The authors repeated the PCR assay. The result is shown below.

**Figure** **2**: Figure 2B contained a gel image with spliced lanes. The results were in 2 gels as shown in the raw data, because there were too many samples. The fermentation of Figure 2 has been repeated and the results have been revised. In Figure 2A the results for “Induction time (h)” are replaced. In the revised Figure 2B the growth curve and the SDS‐PAGE are replaced with the result of the repeated experiment.

Based on the repeated fermentation, the text in the results “3.2 Optimization of Peptibody production from flask to 10‐L bioreactor”, last paragraph, needs to be adjusted and now reads “The time‐course profiles of cell growth and analysis of Peptibody expression were presented in Figure 2B, with a final WCW of 101.98 g/L and an expression level exceeding 20% of total cell protein.”.

**Figure** **4**: Figure 4 contained multiple gel images with sliced lanes, because there were too many samples, and the results were in 2 pieces of gels as shown in raw data. The assays of large‐scale purification have been repeated and the figure has been revised. The graphs of the purification course operated in ÄKTA™ Explorer 100 have been omitted in the revised version as the machine was not available.

**Figure** **7**: The Western Blot in Figure 7C contained spliced bands. This was due to wrong and inconsistent load sequence and subsequent adjustments. In addition, the Akt control or GAPDH loading control did not show the same slicing pattern. The experiment was repeated by the authors. Thus, the text in the Materials and Methods “Cell viability and migration”, line 7, needs to be adjusted and now reads “LL‐2 cells (3 × 10^4^/well) suspended in serum‐free….”. The revised figure is given below.

